# Supplementary material for: Evidence for Weyl fermions in a canonical heavy-fermion semimetal YbPtBi
Source: Nat Commun. 2018 Nov 5;9:4622. doi: 10.1038/s41467-018-06782-1 (PMC6218469; doi:10.1038/s41467-018-06782-1)
Supplement: Supplementary file 1 — Supplementary Information [file 41467_2018_6782_MOESM1_ESM.pdf]

Evidence for Weyl fermions in a canonical heavy-fermion semimetal YbPtBi

Guo et al.

Supplementary Figures

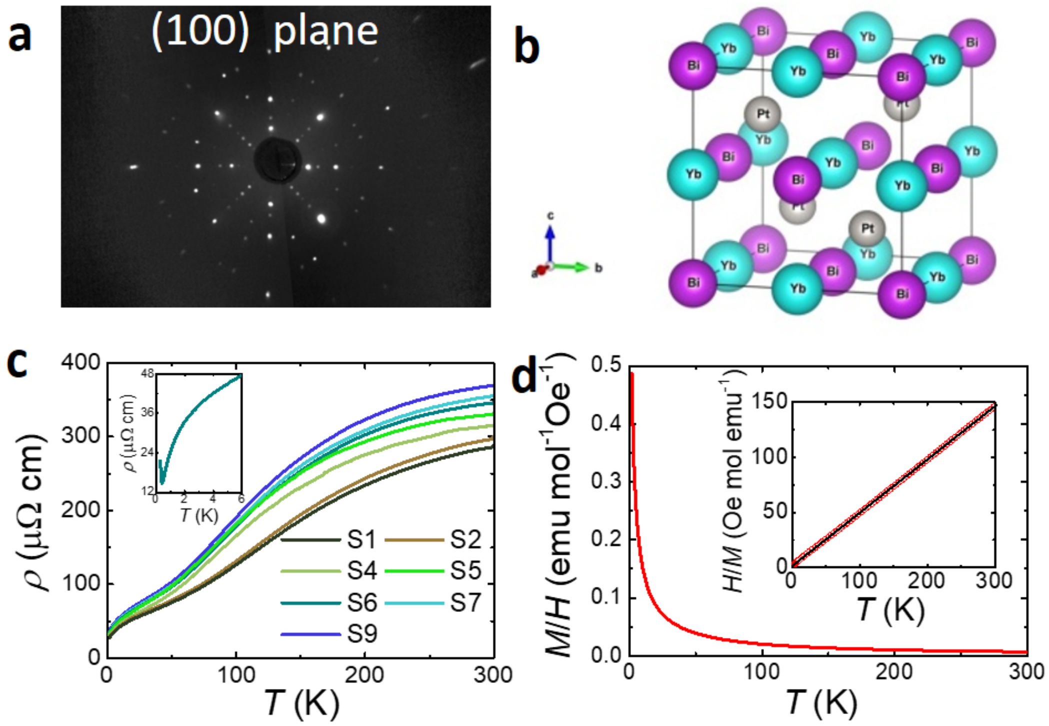

Supplementary Figure 1. Sample characterization. **a**, Laue image of a YbPtBi single crystal along the [100] direction. **b**, Crystal structure of YbPtBi. **c**, Temperature dependence of the resistivity of several samples of YbPtBi at $B = 0$, with the low temperature behavior down to 0.3 K shown in the inset. **d**, Temperature dependence of the magnetic susceptibility of YbPtBi for a field of 0.1 T applied along [100]. The inset shows the inverse susceptibility fitted with Curie-Weiss behavior between 10 and 300 K.

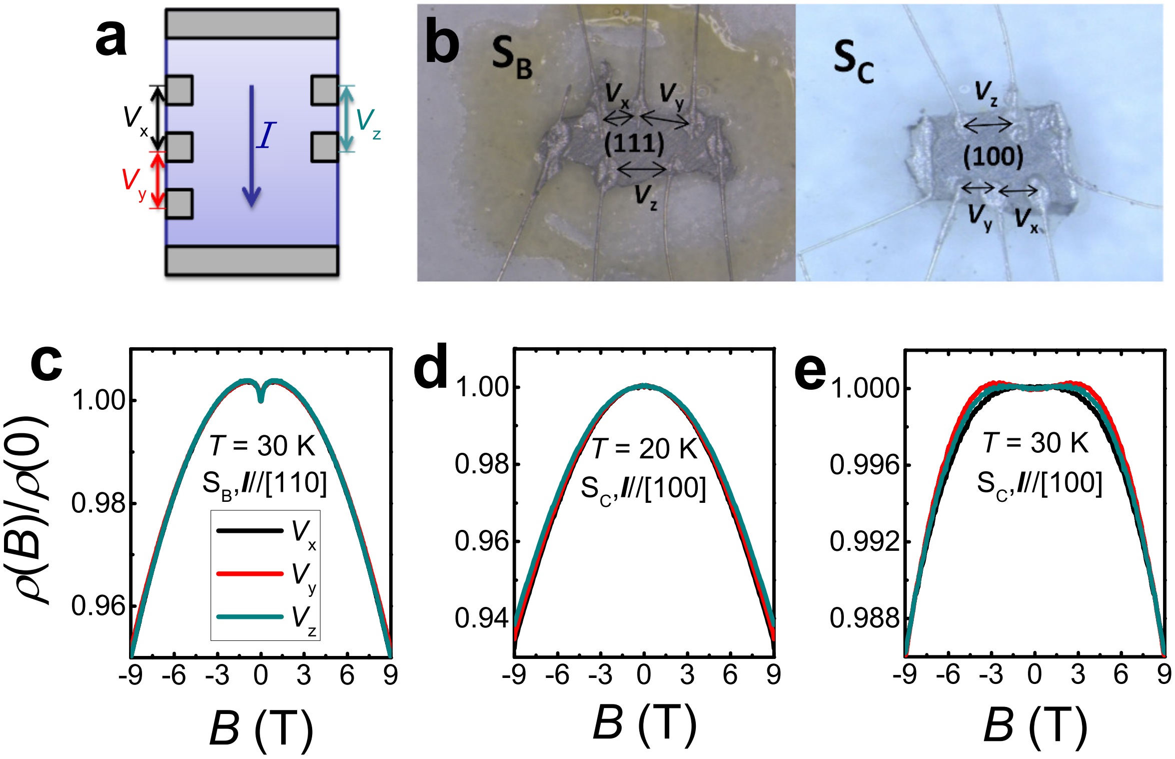

Supplementary Figure 2. Current homogeneity and lack of current jetting. **a**, Illustration of the setup for checking the current homogeneity of resistivity measurements. **b**, Photographs of two samples used to check for current jetting in magnetoresistance measurements, where five voltage wires were attached to the sample, and measurements were made across V_x, V_y and V_z as depicted. The longitudinal magnetoresistivity was measured taking the voltage drop across the three different positions for **c**, S_B at 30 K, and sample S_C at **d**, 20 K and **e**, 30 K.

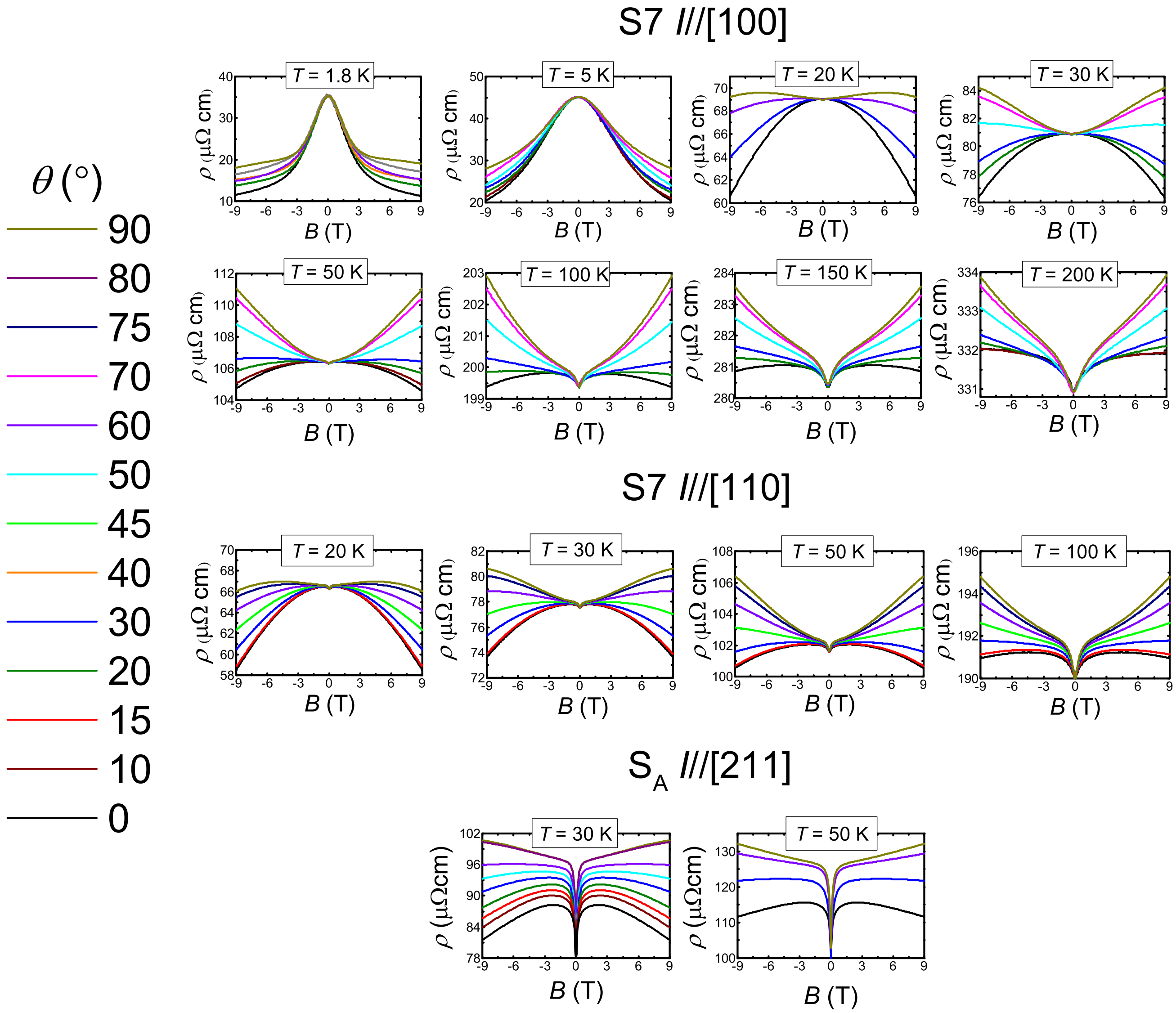

Supplementary Figure 3. Magnetoresistivity for different current orientations. Magnetoresistivity at various temperatures for different angles θ between the current and applied field for measurements with three different current orientations.

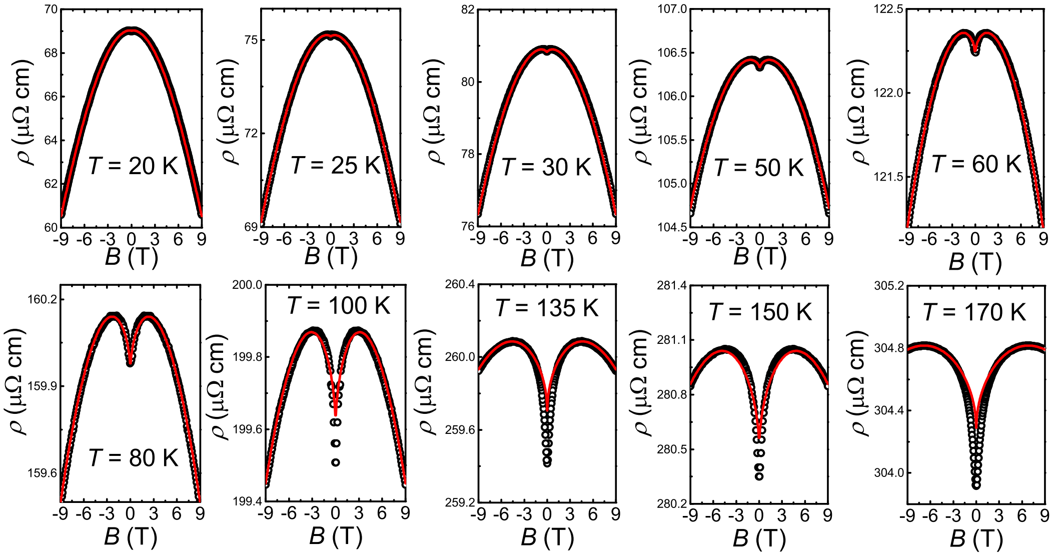

Supplementary Figure 4. Longitudinal magnetoresistivity analysis. The results of fitting the longitudinal magnetoresistivity of sample S7 from 20 K to 170 K using Supplementary Eq. 1. The data and fitting for this sample at selected temperatures are displayed in the main text.

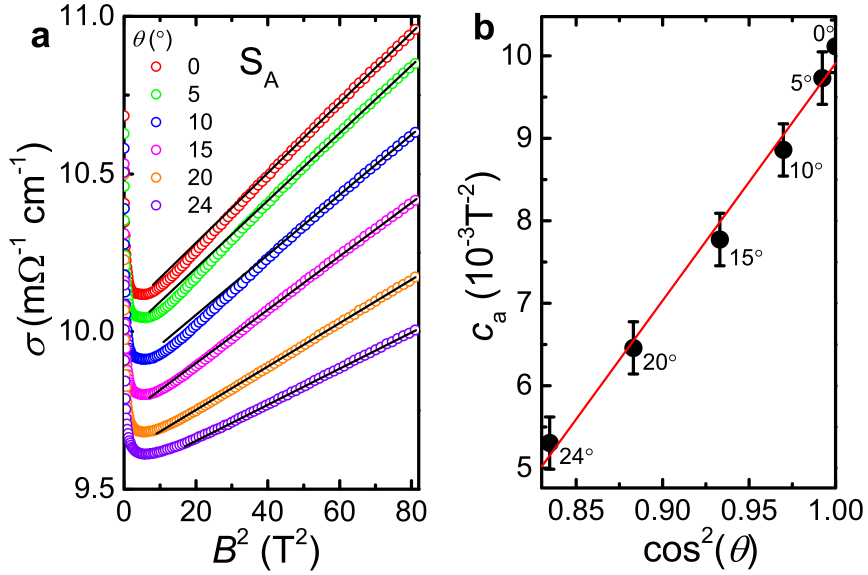

Supplementary Figure 5. Field dependence of the conductivity. a, Field dependence of the conductivity of sample S_A for different angles θ between the field and current, for a current along [211].**b**, The angle dependence of the chiral constant c_a for the same sample, demonstrating that $c_a \propto \cos^2\theta$. The values of c_a are determined from fitting the conductivity using linear regression and the error bars are the corresponding standard errors.

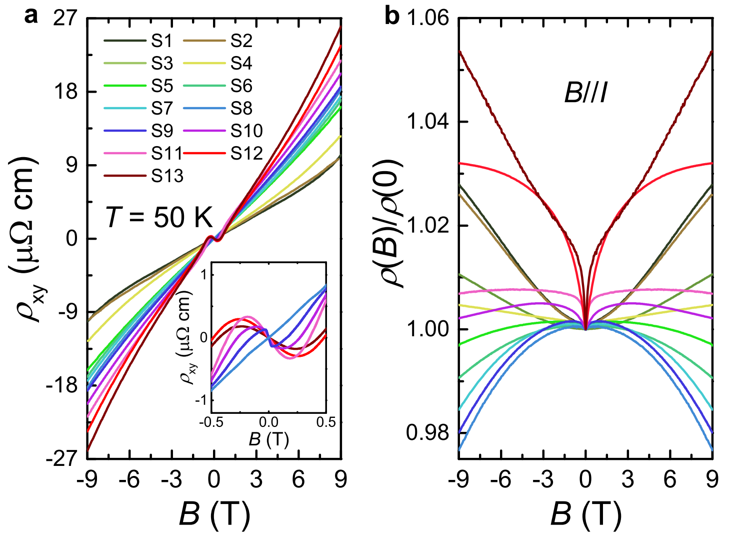

Supplementary Figure 6. Hall resistivity at 50 K. a, Magnetic field dependence of the Hall resistivity at 50 K for various samples of YbPtBi from several batches, where the low field data is displayed in the inset. **b**, The corresponding magnetic field dependence of the longitudinal magnetoresistance at 50 K.

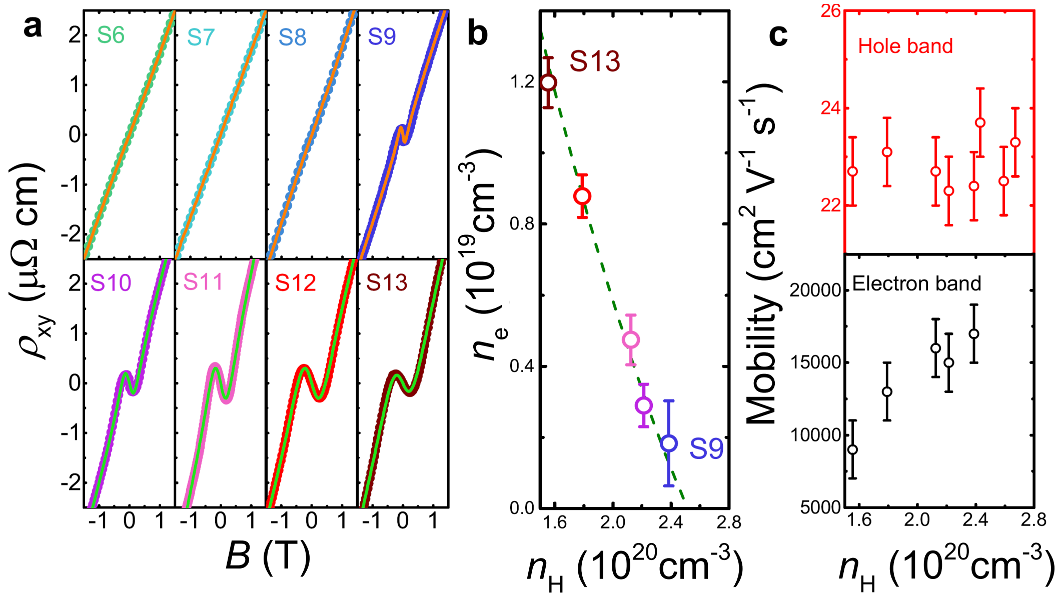

Supplementary Figure 7. Analysis of the Hall resistivity at 50 K. a, Magnetic field dependence of the Hall resistivity for various samples at 50 K, where the samples S1-S8 were fitted with a single-band model, while S9-S13 were fitted with two-bands, and the anomalous contribution from the magnetization is taken into account. The fitted **b**, carrier densities, and **c**, mobilities, for the two band fits to the Hall resistivity. The data were fitted using a least squares method, with the error bars corresponding to the standard errors of the fitted parameters.

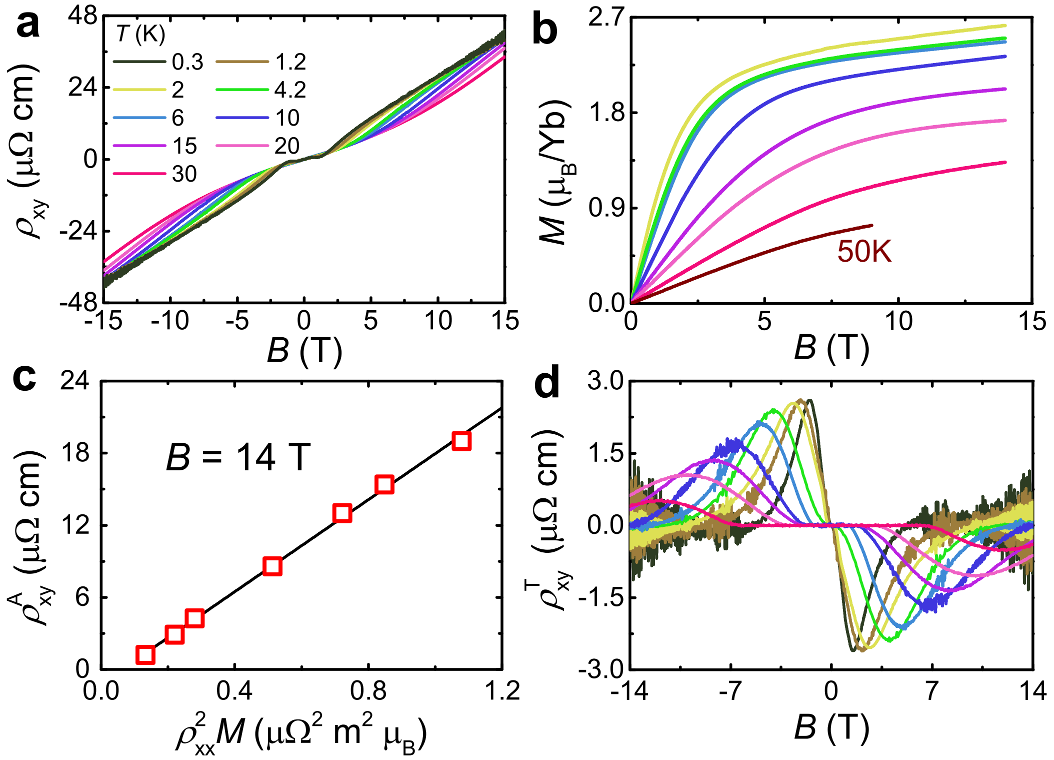

Supplementary Figure 8. Analysis of the anomalous Hall effect. **a**, Field dependence of the Hall resistivity of sample S6 at various temperatures from 0.3 to 30 K, for magnetic fields along [100]. **b**, The field dependence of the magnetization along the same direction at the corresponding temperatures. **c**, The component of the anomalous Hall effect arising due to the magnetization $\rho_{\rm xy}^{\rm A}$ at each temperature, as a function of $\rho_{\rm xx}^2 M$ at 14 T, where there is a clear linear relationship. **d**, Topological contribution to the anomalous Hall resistivity $\rho_{\rm xy}^{\rm T}$, which shows a clear peak which moves to higher fields with increasing temperature.

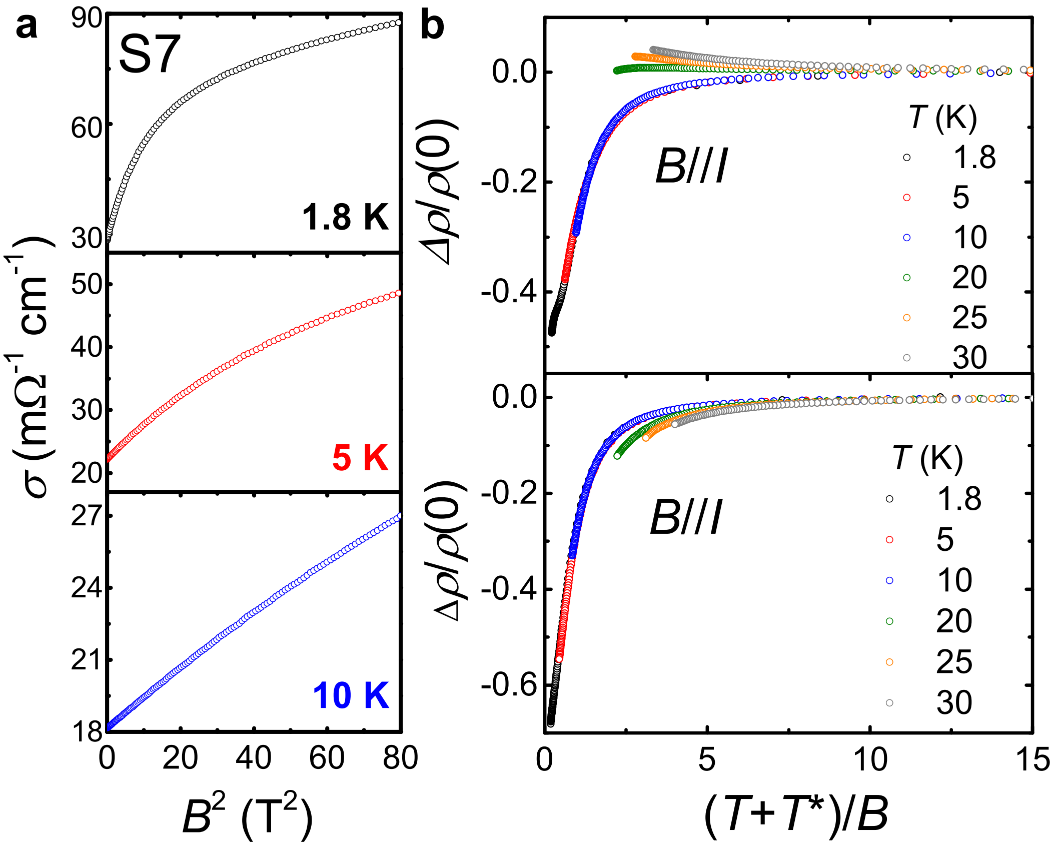

Supplementary Figure 9. Low temperature conductivity in longitudinal and transverse fields. **a**, Low temperature conductivity of sample S7 at 1.8 K, 5 K and 10 K up to a maximum applied field of 9 T, which do not show the B^2 dependence expected from the chiral anomaly contribution. This is despite the evidence for the chiral anomaly in this sample from measurements above 20 K (Fig. 2 and Supplementary Fig. 4) **b**, Scaling of the magnetoresistance using the behavior of a single ion Kondo impurity model for applied fields perpendicular, and parallel to the current [10]. Below 20 K the data for both field directions overlap well, indicating the dominance of Kondo scattering and the absence of any significant contribution from the chiral anomaly. This scaling fails at $T \geq 20$ K.

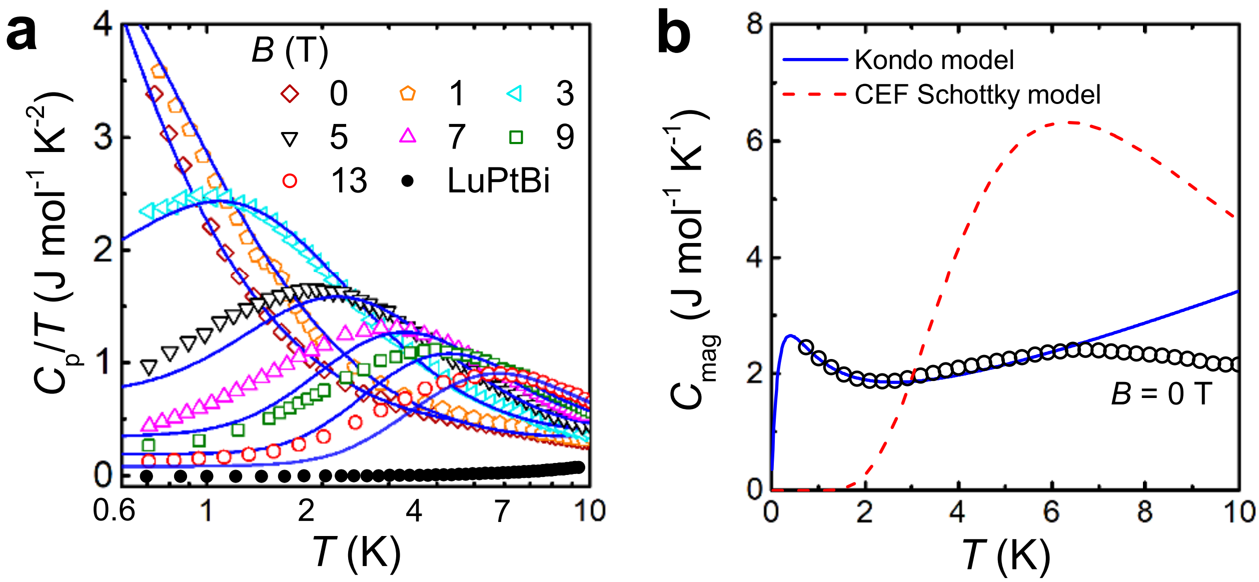

Supplementary Figure 10. Specific heat in applied fields. **a**, The temperature dependence of the specific heat at various fields fitted with the Kondo model (Supplementary Eq. 5). **b**, Magnetic contribution to the temperature dependent specific heat of YbPtBi after subtracting the phonon contribution. The solid line shows a fit to the Kondo model, while the dashed line shows the calculated contribution from a Schottky model with a quartet 1.45 meV above the ground state doublet.

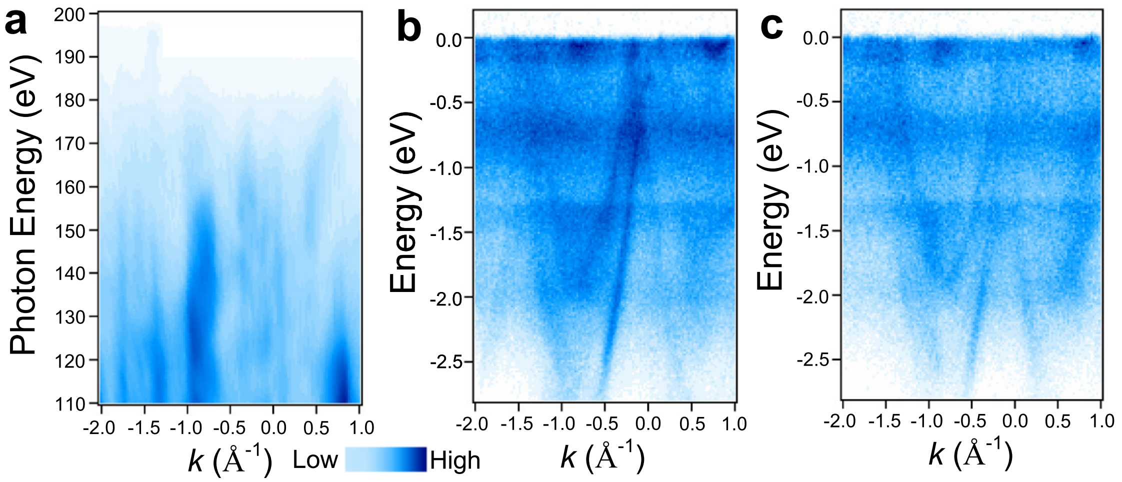

Supplementary Figure 11. Resonant photoemission measurements across the Yb N edge ($4d$ to $4f$ transition). a, Stacks of momentum distribution curves at the Fermi energy, taken with a large photon energy range, including the Yb N-IV and N-V edges between 180 and 190 eV. No resonance contrast can be observed, indicating that most of the Yb f bands are filled (and therefore unavailable for resonant excitation). ARPES spectra are shown taken at **b**, 175 eV (off resonance) and **c**, 185 eV (on resonance) along the $\bar{\Gamma}\bar{M}$ direction. While no resonance enhancement of photoemission intensity can be observed, we do observe bulk f-bands lying near the Fermi level, and the spin-orbit split satellite peak at -1.2 eV. The flat f-bands at -0.8 and -2 eV are derived from surface Yb layers, which have f electron energies different from the bulk. The states near the point $k_x \approx 0.8$ Å^{-1} at the Fermi level are due to surface states on a Bi terminated YbPtBi(111) surface. Due to the suppression of photoemission intensity of the electron pocket at this high photon energy, as well as strong emission from f-bands, the fine features near the triply degenerate fermion points cannot be well resolved here. Note that the bulk Yb f bands near the Fermi level are only observed for photon energies $h\nu > 100$ eV and soft x-ray measurements (see Supplementary Figure 12). This is consistent with the general understanding that the photoexciation cross section for f orbitals is small at low photon energies. This could potentially make it difficult to use laser or pump-probe ARPES with small photon energies to probe Weyl fermions in the heavy fermions state.

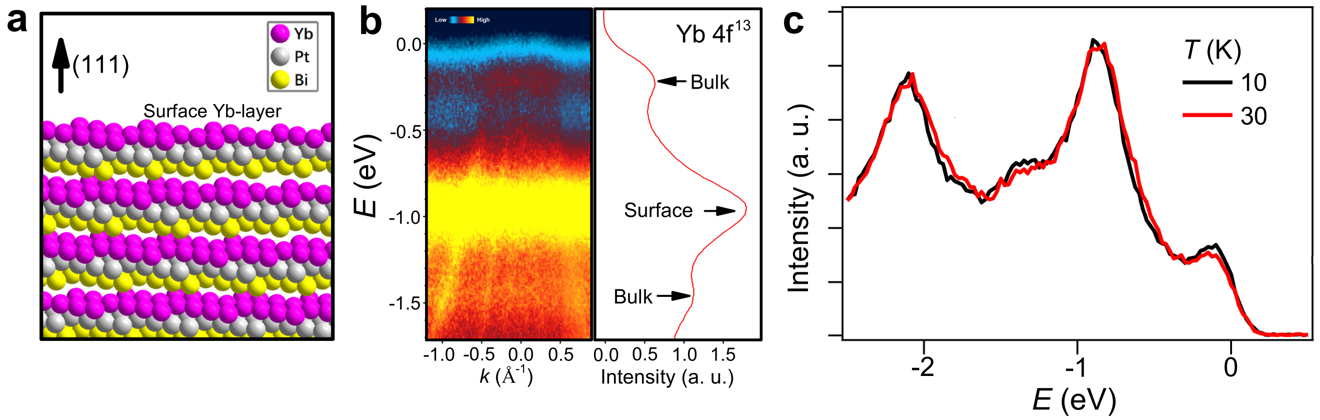

Supplementary Figure 12. Soft X-ray ARPES measurements revealing the bulk f-band in YbPtBi. **a**, Layering structure of the Yb-terminated YbPtBi (111) surface. Since the bulk-like Yb layer lies beneath the surface, Yb, Pt and Bi layer, only high photon energy ARPES is able to detect the bulk f-signal. **b**, Soft x-ray ARPES results (at 760 eV) of YbPtBi at 10 K, revealing the bulk f-band near the Fermi level. At the crossing between the f bands and valence bands, weak distortion in the dispersion can be identified, implying possible $f - c$ hybridization. The electron pocket is not observed here due to weak photoemission intensity at high photon energies and the dominant emission from the f bands. **c,** Temperature dependence of the energy distribution curve, revealing a small decrease of the f band near E_{F} at higher temperatures (the same is true for the spin-orbit split satellite at -1.3 eV), implying that there might already be a possible contribution from the Kondo resonance at 10 K, consistent with our transport measurements. The peaks at -0.8 and -2.2 eV are from surface Yb layers, which do not show an obvious temperature dependence.

Supplementary Tables

$\mu_0 H$(T)	v^*(ms^{-1})	γ(mJ mol^{-1} K^{-2})	$\Delta/k_{\rm B}$ (K)	$T_{\rm Kondo}^{\rm min}$(K)	$T_{\rm Weyl}^{\rm max}$(K)
0	–	–	0.9	–	–
1	–	–	1.1	–	–
3	–	–	1.3	1.0	–
5	–	–	1.6	2.4	–
7	213	244	1.7	3.7	1.0
9	292	182	2.6	4.3	1.9
13	394	89	3.7	5.0	2.9

Supplementary Table 1. Results of fitting the specific heat of YbPtBi. The parameter Δ which characterizes the resonance width in the Kondo resonance level model is obtained from fitting Supplementary Eq. 5. The effective Fermi velocity v^* and Sommerfeld coefficient γ are obtained from fitting the Weyl node term in the specific heat in Supplementary Eq. 7. The temperature up to which there is a T^3 contribution to the specific heat from the Weyl nodes ($T_{\rm Weyl}^{\rm max}$) and the temperature above which the Kondo resonance level model is well fitted ($T_{\rm Kondo}^{\rm min}$(K)) are also displayed.

Supplementary Notes

Supplementary Note 1: Current jetting

A possible origin of an observed negative longitudinal magnetoresistance in materials with a large transverse positive magnetoresistance is the current jetting effect [2]. This has been found to occur in materials with a large resistivity anisotropy A, where A is the ratio of the transverse and longitudinal resistivities. This can lead to an inhomogenous current distribution in the sample, especially when A is greater than l/w, the ratio of the length between the voltage wires and the sample width. While the value of A is very large for some Weyl semi-metal candidates, the anisotropy of YbPtBi is rather small, for instance in sample S7 the largest measured value in 9 T is $A = 1.6$ at 2 K. Furthermore at higher temperatures, once the transverse magnetoresistance becomes positive, A is further reduced and is only 1.06 at 50 K. Therefore current jetting cannot be the origin of the negative magnetoresistance. To further rule out an inhomogenous current, for samples $\mathrm{S_B}$ and $\mathrm{S_C}$, five voltage wires were attached to the sample and the voltage across three positions were measured, as illustrated in Supplementary Fig. 2a and shown in Supplementary Fig. 2b. As displayed in Supplementary Figs. 2c-2e, very similar magnetoresistances are measured across the three different positions, showing that there is a highly homogenous current and that current jetting does not affect the measurements.

Supplementary Note 2: Magnetoresistance fitting

The field dependence of the resistivity is displayed in Supplementary Fig. 3 for measurements with three different current directions. It can be seen that in all cases, above 20 K there is a negative longitudinal magnetoresistance at 9 T, which weakens upon increasing the angle θ between the field and current, before eventually becoming positive. This is evidence for the chiral anomaly, which is further supported by the fact that this observation does not depend on the direction of the current with respect to the crystal axes, only the angle θ. It can also be seen that at low temperatures below 20 K, the magnetoresistance is negative along all directions, which is attributed to Kondo scattering.

The longitudinal magnetoresistance was fitted using a conductivity given by

$$\sigma(B) = (1 + c_{\mathrm{a}}B^2)\sigma_{\mathrm{WAL}} \tag{1}$$

where c_{a} is the chiral constant giving the strength of the chiral anomaly contribution and σ_{WAL} contains the contribution from the weak antilocalization, where

$$\sigma_{WAL} = \sigma_{\mathrm{N}} + a\sqrt{B} \tag{2}$$

The longitudinal magnetoresistance could be fitted from 20 K to 170 K using Supplementary Equation 1 and the fitting results are displayed for sample S7 in Supplementary Fig. 4. The data are well fitted, indicating a significant contribution of the chiral anomaly in this temperature range. At 200 K the longitudinal magnetoresistance is positive, while at 10 K the data cannot be fitted and

the magnetoresistance is negative for all θ. In this low temperature region the contribution from the incoherent Kondo scattering dominates and the component from the chiral anomaly is negligible, as shown by the scaling behavior described below. As displayed in the main text, c_{a} of sample S7 shows the $\sim \cos^2\theta$ dependence expected from the Weyl node contribution. In Supplementary Fig. 5 the field dependence of the conductivity and angular dependence of c_{a} are also displayed for sample $\mathrm{S_A}$, where the current is along the [211] direction. It can be seen that much like sample S7, the conductivity $\sigma \propto B^2$ at high fields and c_{a} shows a $\propto \cos^2\theta$ dependence, demonstrating that this behavior is reproducible upon changing the current orientation.

Supplementary Note 3: Relationship between the carrier density and magnetoresistance

Supplementary Fig. 6 shows the field dependence of the Hall resistivity and longitudinal magnetoresistance at 50 K for samples from various batches. Due to the zero gap semiconducting nature of many half Heusler compounds, the carrier densities are readily tunable [3, 4], and in the case of YbPtBi we find that by increasing the fraction of Bi used during the sample synthesis, samples with a larger hole carrier density (n_{H}) can be obtained, while partial substitution of Pt by Au (for samples S11-S13) yields more electron doped samples. As shown by the inset of Supplementary Fig. 6a, some samples (S9-S13) show a hump at low fields, which indicates two band behavior, with one hole and one electron band, as found previously [5]. The data for samples S1-S8 were fitted with single (hole) band behavior, while S9-S13 which have this low field feature were fitted with two-bands. An anomalous Hall contribution proportional to the magnetization M, $\rho_{\mathrm{xy}}^{\mathrm{A}} \propto \rho_{\mathrm{xx}}^2 M$ (Supplementary Fig. 8c), was also taken into account, and the fitted curves at 50 K consisting of the ordinary band part and anomalous contribution are displayed in Supplementary Fig. 7a, with the fitted carrier densities and mobilities from the two-band fits shown in Supplementary Figs. 7b and 7c respectively.

Supplementary Note 4: Anomalous Hall effect

So as to analyze the anomalous Hall effect, sample S6 which exhibits single band behavior in the Hall resistivity (Supplementary Fig. 6a) was measured at lower temperatures, from 0.3 K to 30 K, as displayed in Supplementary Fig. 8a. The total Hall resistivity can be written as [6]

$$\rho_{\mathrm{xy}} = \rho_{\mathrm{xy}}^{\mathrm{N}} + \rho_{\mathrm{xy}}^{\mathrm{A}} + \rho_{\mathrm{xy}}^{\mathrm{T}} \tag{3}$$

where $\rho_{\mathrm{xy}}^{\mathrm{N}}$, $\rho_{\mathrm{xy}}^{\mathrm{A}}$, and $\rho_{\mathrm{xy}}^{\mathrm{T}}$ are the normal Hall effect, anomalous term from the magnetization, and the topological Hall effect term respectively [7]. Since the carrier density changes with temperature [1], we approximate $\rho_{\mathrm{xy}}^{\mathrm{N}}$ by fitting the Hall resistivity with a single band model in the high field range where the magnetization is nearly constant, and this was subtracted from the data to yield the anomalous contribution. The term from the magnetization $\rho_{\mathrm{xy}}^{\mathrm{A}}$ is given by

$$\rho_{\mathrm{xy}}^{\mathrm{A}} = \alpha\rho_{\mathrm{xx0}}M + \beta\rho_{\mathrm{xx0}}^2 M + b\rho_{\mathrm{xx}}^2 M, \tag{4}$$

which correspond to the skew scattering, side-jump and intrinsic contributions respectively [8], where ρ_{xx0} is the residual resistivity. Importantly, since all these terms are proportional to the magnetization M (displayed in Supplementary Fig. 8b), ρ_{xy}^{A} can be estimated by scaling the magnetization onto the AHE data, as shown by the dashed lines in Fig. 4a of the main text. It can be seen in Supplementary Fig. 8c that the ρ_{xy}^{A} at different temperatures scale linearly as a function of $\rho_{xx}^{2}M$ at 14 T, giving a clear indication that the estimation of ρ_{xy}^{A} is reliable, and that the intrinsic contribution dominates. It is noted that for the analysis of the data at 0.3 K and 1.2 K, $M(H)$ at 2 K was utilized as an approximation, but due to the rapid decrease of resistivity at low temperatures, ρ_{xy}^{A} is negligible here, with very little contribution to ρ_{xy}. After subtracting ρ_{xy}^{A}, the topological Hall effect term ρ_{xy}^{T} can be obtained, and is displayed in Supplementary Fig. 8d. A clear peak is observed in ρ_{xy}^{T} as a function of field, which shifts to higher field as the temperature increases. Meanwhile the maximum value at 0.3 K corresponds to a very large topological Hall angle $\Theta_{xy}^{T} = \Delta\sigma_{xy}^{T}/\sigma_{xx}$ of 0.18, which is very similar to the value of 0.17 in the magnetic Weyl semimetal GdPtBi [6]. Meanwhile another large value of around 0.4 was found in another magnetic Weyl fermion system Mn_3Sn [9].

Supplementary Note 5: Disappearance of the chiral anomaly in the magnetoresistance

Very different behavior in the magnetoresistance is found below 20 K, where as shown in Supplementary Fig. 9a, the conductivity $\sigma(B)$ does not display a B^2 dependence and the magnetoresistance is negative for *all* θ. Here, the negative magnetoresistance is ascribed to Kondo scattering, and as shown in Supplementary Fig. 9b, the low temperature curves as a function of $(T + T^*)/B$ nicely overlap for $T^* = 0.8$ K. Such a scaling was predicted on the basis of a single impurity Kondo model [10], and has also been observed in other Yb based heavy fermion materials [11, 12]. This indicates that at low temperatures the magnetoresistance can be accounted for by Kondo scattering and the chiral anomaly contribution at very low temperatures is negligible.

Supplementary Note 6: Specific heat fitting of YbPtBi

The temperature dependence of the specific heat as C/T of YbPtBi in various applied fields up to 13 T is displayed in Supplementary Fig. 10. The data were first fitted using the expression

$$C = \gamma_0 T + \beta T^3 + C_{\mathrm{Kondo}}(H, T) \tag{5}$$

where γ_0 is the residual Sommerfeld coefficient, β corresponds to the phonon contribution βT^3 fixed from the value for LuPtBi, while C_{Kondo} is the specific heat calculated within a Kondo resonance level model for an effective $S = \frac{1}{2}$ given by [13]

$$\frac{C_{\mathrm{Kondo}}}{T} = \frac{\Delta}{\pi T^2} - \frac{2k_{\mathrm{B}}}{T} Re\left\{\frac{(\Delta + ig\mu_{\mathrm{B}}H)^2}{(2\pi k_{\mathrm{B}}T)^2}\left[4\psi'\left(1 + \frac{\Delta + ig\mu_{\mathrm{B}}H}{\pi k_{\mathrm{B}}T}\right) - \psi'\left(1 + \frac{\Delta + ig\mu_{\mathrm{B}}H}{2\pi k_{\mathrm{B}}T}\right)\right]\right\} \tag{6}$$

where ψ' is the derivative of the digamma function, H is the applied field, $g = 2$ is the Landé g factor and Δ is the resonance width, which at sufficiently low fields can be identified as $\Delta = k_{\mathrm{B}}T_{\mathrm{K}}$. The results are shown in Supplementary Fig. 10a, where while this model can reasonably well account for the 0 and 1 T data at lower temperatures, at higher fields there is now a clear low temperature deviation, with a contribution to C/T not taken into account by Supplementary Eq. 5. This low temperature behavior is well fitted by the Weyl node contribution [14]

$$C = \gamma T + \frac{7\pi^2 k_{\mathrm{B}}}{30}\left(\frac{k_{\mathrm{B}}T}{\hbar v^*}\right)^3 \tag{7}$$

where v^* is the effective Fermi velocity. The fitted parameters for both models are displayed in Supplementary Table 1. The temperatures above which the Kondo resonance level model can be fitted, as well as the maximum temperature fitted with a T^3 dependence are also displayed. In an applied field of 5 T, there was not enough data at low temperatures below the peak to fit to Supplementary Eq. 7, but it could be clearly fitted for data at 7, 9 and 13 T. The increase of v^* and the decrease of γ are all consistent with the decrease of quasiparticle mass in field. The magnetic contribution in zero field obtained by subtracting the phonon contribution is displayed in Supplementary Fig. 10b. It can be seen from the solid line that Supplementary Eq. 5 can well account for the data at low temperatures and shows reasonable agreement up to around 6 K with $T_{\mathrm{K}} = 0.9$ K and $\gamma_0 = 0.31$ J/mol K^2. At around 6.5 K there is a weak maximum in the magnetic specific heat, which was previously ascribed to a crystalline-electric field (CEF) excitation [1]. The CEF scheme for YbPtBi has not been unambiguously resolved, where inelastic neutron scattering measurements are consistent with a ground state Γ_7 doublet, with the first excited level being a Γ_8 quartet at less than 2 meV and the second excited level being a Γ_6 doublet at 6 meV [15]. Therefore it could be that this peak corresponds to the Γ_8 level, but a simple two-level Schottky model which matches the peak position with a gap of $\Delta_{\mathrm{Schottky}} = 1.45$ meV vastly overestimates the experimental data, as shown by the dashed line. This is consistent with the significant broadening of the Γ_8 level inferred from the neutron scattering results [15]. These results therefore suggest that the low temperature data will not be dominated by the CEF excitations.

Supplementary References

[1] Mun, E. D. *et al.* Magnetic-field-tuned quantum criticality of the heavy-fermion system YbPtBi. *Phys. Rev. B* **87**, 075120 (2013).

[2] dos Reis, R. D. *et al.* On the search for the chiral anomaly in Weyl semimetals: the negative longitudinal magnetoresistance. *New J. Phys.* **18**, 085006 (2016).

[3] Chadov, S. *et al.* Tunable multifunctional topological insulators in ternary Heusler compounds. *Nature Materials* **9**, 541–545 (2010).

[4] Hirschberger, M. *et al.* The chiral anomaly and thermopower of Weyl fermions in the half-Heusler GdPtBi. *Nature Materials* **15**, 1161–1165 (2016).

[5] Schilling, M. B. *et al.* Two-channel conduction in YbPtBi. *Phys. Rev. B* **95**, 155201 (2017).

[6] Suzuki, T. *et al.* Large anomalous Hall effect in a half-Heusler antiferromagnet. *Nature Physics* **12**, 1119 (2016).

[7] Manna, K., Sun, Y., Müchler, L., Kübler, J. & Felser, C. Heusler, Weyl, and Berry. *Nature Reviews Materials* **3**, 244–256 (2018).

[8] Li, Y. *et al.* Robust formation of skyrmions and topological Hall effect anomaly in epitaxial thin films of MnSi. *Phys. Rev. Lett.* **110**, 117202 (2013).

[9] Li, X. *et al.* Momentum-space and real-space Berry curvatures in Mn_3Sn. *arXiv:1802.00277* (2018).

[10] Schlottmann, P. Some exact results for dilute mixed-valent and heavy-fermion systems. *Phys. Rep.* **181**, 1–119 (1989).

[11] Diehl, J. *et al.* Magnetic properties of the heavy fermion antiferromagnets YbNiAl and YbPtAl. *Physica B: Condensed Matter* **206**, 344 – 348 (1995).

[12] Pietri, R., Andraka, B., Kaczorowski, D., Leithe-Jasper, A. & Rogl, P. Magnetoresistance and low-temperature specific heat of the Yb compounds YbRhSn, YbPdBi, and YbPtSn. *Phys. Rev. B* **61**, 12169–12173 (2000).

[13] Schotte, K. & Schotte, U. Interpretation of Kondo experiments in a magnetic field. *Phys. Lett. A* **55**, 38 – 40 (1975).

[14] Lai, H.-H., Grefe, S. E., Paschen, S. & Si, Q. Weyl–Kondo semimetal in heavy-fermion systems. *Proceedings of the National Academy of Sciences* **115**, 93–97 (2018).

[15] Robinson, R. A. *et al.* Low-energy excitations and the electronic specific heat of YbBiPt. *Phys. Rev. Lett.* **75**, 1194–1197 (1995).

[16] Fujimori, S.-i. Band structures of 4f and 5f materials studied by angle-resolved photoelectron spectroscopy. *J. Phys. Condens. Matter* **28**, 153002 (2016).
